# ComBat Harmonization With and Without Empirical Bayes Estimation for Resting-State Functional Connectivity in Pediatric Mild Traumatic Brain Injury: a CARE4Kids Study

**DOI:** 10.21203/rs.3.rs-9418750/v1

**Published:** 2026-05-06

**Authors:** Adrian I Onicas, Finian Keleher, Kevin C Bickart, Christine L Mac Donald, Anne Brown, Lawrence Cook, Frederick P Rivara, Gerard A Gioia, Christopher C Giza, Emily L Dennis

**Affiliations:** University of Utah School of Medicine; University of Utah School of Medicine; University of California, Los Angeles; University of Washington School of Medicine; University of California, Los Angeles; University of Utah School of Medicine; University of Washington and Seattle Children’s Research Institute; Children’s National Hospital and George Washington University School of Medicine; University of California, Los Angeles; University of Utah School of Medicine

**Keywords:** multi-site neuroimaging, resting-state fMRI, functional connectivity, ComBat harmonization, empirical bayes, mild traumatic brain injury

## Abstract

ComBat is commonly used to mitigate batch effects in multi-site neuroimaging studies. Although empirical bayes estimation is a key component of ComBat, its impact on resting-state functional connectivity measures, including site effect reductions and preservation of original within-site variability remains underexplored. Empirical bayes estimation was evaluated on functional connectivity in adolescents with mild TBI. Resting-state fMRI data were collected from 144 adolescents across six sites participating in the CARE4Kids study. Functional connectivity was computed over resting state networks defined using Seitzman parcellation. ComBat with and without empirical bayes estimation was assessed by quantifying: (1) site variability based on one-way ANOVA, (2) within-site consistency before and after harmonization (intraclass correlation coefficient), (3) age correlations, and (4) site effects of the principal components. All networks demonstrated significant moderate-to-large site effects (all *p* < 0.001) before harmonization. Harmonization without empirical bayes yielded low residual site effect sizes, whereas empirical bayes harmonization yielded low-to-medium site effects. Within-site consistency between the data before and after harmonization was consistently excellent when using empirical bayes estimation. Harmonization without empirical bayes was excellent across sites with higher sample sizes (n ≥ 13), but moderate-to-excellent for smaller sites (n ≤ 12). Both approaches improved age associations across connections involving the default mode and dorsal attention networks and reduced site effects in the first principal component (*p* > 0.05). ComBat with or without empirical bayes effectively removes site variability while preserving age in functional connectivity measures, however, empirical bayes estimation better preserves the original within-site variability.

## 1 Introduction

Neuroimaging research in pediatric concussion has been constrained by reliance on small, heterogeneous samples, which have contributed to variability and inconsistencies in reported outcomes [1, 2]. Multi-site neuroimaging studies have the potential to address these challenges by involving larger sample sizes with enhanced statistical power, which may improve reproducibility, and enable the identification of neurobiological alterations associated with concussion in developing brains [3, 4]. Site-specific variations in imaging protocols and scanner characteristics can introduce batch effects, potentially confounding biological findings and hindering the generalizability of connectivity analyses derived from resting-state functional MRI (rs-fMRI) [5]. Standardization efforts, such as processing pipelines that enforce uniform preprocessing across sites, help mitigate some sources of variability, but additional statistical harmonization techniques are necessary to account for residual scanner-related effects [6]. Correcting for site-related variability is necessary to ensure reliable functional connectivity measures, particularly in heterogeneous cohorts such as individuals with traumatic brain injury (TBI).

Various harmonization methods have been developed, with ComBat emerging as a widely adopted approach in neuroimaging research [7]. Originally developed for genomic data [8], ComBat has been successfully adapted for neuroimaging applications, demonstrating its efficacy in accounting for site effects while preserving biological variability associated to age [7, 9]. A key feature of the ComBat algorithm is the Empirical Bayes (EB) estimation, which pools information across features to improve estimation of batch effect parameters [8]. Specifically, ComBat uses EB to estimate scanner-specific location and scale parameters for each feature in the dataset. Location adjustment involves correcting for differences in the mean of features across different batches, addressing additive batch effects. Scale adjustment focuses on correcting for differences in the variance or spread of features across batches, targeting multiplicative batch effects. Subsequently, ComBat leverages the EB framework to pool information across all features, thereby refining the estimation of these parameters across different sites [7]. ​​When the EB=FALSE option is used, ComBat functions as a univariate location and scale (L/S) adjustment model, performing L/S adjustments independently for each feature [7].

The rationale of EB estimation is to stabilize site effect estimates by leveraging information across features, which can be particularly beneficial for batches with low variability or sparse data. ComBat documentation recommends using EB when the number of features is higher than the sample size, and using feature-specific adjustments (i.e., EB=FALSE) otherwise [10]. While a higher number of features does not imply that sufficient commonality in the batch effects is met to facilitate effective information pooling, average within- and between-network connectivity measures, commonly employed to study brain function after mild TBI [11, 12], tend to have similar mean and variance across features. This suggests EB estimation could be appropriate even when the number of features is smaller than the sample size. The influence of EB estimation on ComBat harmonization likely depends on the characteristics of the data [8, 9], and EB estimation has been widely applied and tested for features derived using structural neuroimaging in previous ComBat studies [7, 9, 13]. However, its use in functional connectivity remains limited, especially in special populations with heterogeneous data such as pediatric mild TBI. Previous work has raised concerns that multisite harmonization could alter original variability present in structural neuroimaging features, potentially removing meaningful within-site variability [14]. While the adaptation of ComBat for neuroimaging allows explicit inclusion of additional covariates to preserve biological variability, incorporating complex variables that contribute to site differences unrelated to batch effects can be challenging. Variability between sites may reflect genuine differences in participant characteristics beyond site effects, which may mirror more complex biological, psychological, or demographic characteristics, or heterogeneity of concussion. Assessing within-site consistency can help identify harmonization approaches that better preserve meaningful variability without compromising the removal of site effects.

The current study addresses this gap by evaluating ComBat harmonization for within- and between-network functional connectivity measures in a pediatric mild TBI cohort. Specifically, the objective of this study is to evaluate the impact of using the EB estimation in ComBat harmonization on resting-state functional connectivity measures in adolescents with mild traumatic brain injury. We aim to assess how the use of EB affects: (1) the effectiveness of ComBat in reducing site-related variability for individual connectivity measures, (2) the preservation of original within-site variability, and (3) the ability to address multivariate site effects, as evaluated by examining the presence of site-related variance in the principal components from within- and between-network functional connectivity measures. We expect that harmonization without EB will more effectively remove site effects and preserve the original variability of individual features, whereas EB harmonization is anticipated to be more efficient at addressing multivariate site effects in the principal components, by leveraging mean and variance estimates across features derived from functional connectivity.

## 2 Methods

### 2.1 Study Design and Procedure

Data were drawn from the CARE4Kids study [3], which employs a longitudinal design to investigate persistent post-concussive symptoms in adolescents. Participants were recruited from emergency departments, primary care clinics, concussion specialty clinics, athletic trainers, and through community outreach. Inclusion criteria include age range between 11 to 17.99 years, a concussion diagnosis by a healthcare provider, and persistence of at least one post-concussion symptom worse than pre-injury baseline within 35 days post-injury. Exclusion criteria include severe developmental delays, significant neurological disorders (with the exception of migraines), severe psychiatric illness, substance abuse, or a history of moderate/severe traumatic brain injury. The study protocol was approved by a central institutional review board at the University of Utah. All participants and their families were provided with detailed study information, ensuring voluntary participation and confidentiality. This multi-center study includes extensive biomarker, neuropsychological, and clinical data collection and was conducted across six consortium sites: University of California, Los Angeles (UCLA), University of Washington/Seattle Children’s Research Institute (SEAT), Children’s National Medical Center (CNMC), University of Texas Southwestern Medical Center (UTSW), University of Rochester (ROCH), and Atrium Health Wake Forest Baptist Hospital (WAKE). Three data collection points (ROCH, SEAT, and UCLA) were encoded with different sites due to a software update that may introduce variations in image acquisition and reconstruction [15, 16]. This resulted in a total of 9 sites for the harmonization analysis (see Table 1).

### 2.2 Image acquisition and preprocessing

Structural T1-weighted (T1w) and resting-state functional brain MRI data were acquired at 3 tesla and were processed using the high-performance computing resources provided by the Center for High-Performance Computing (CHPC) at the University of Utah. Data from the baseline assessment (7 to 35 days post-injury) has been used for the purposes of this analysis. Two rs-fMRI runs were obtained for 5.11 min each with a repetition time of 800 ms (383 volumes per run), voxel size of 2.4 mm^3^, 60 slices, and echo time (TE) of 30 ms. The T1w imaging was performed with a voxel size of 1 mm^3^, 176–225 slices. Batches SEAT1 and SEAT2 had differing voxel sizes of 2.25 × 2.25 × 2.4 mm. The images preprocessed analyzed using HALFpipe (Harmonized AnaLysis of Functional MRI pipeline) version 1.2.2 [17], which is based on fMRIPrep version 20.2.7 [18], following standardized protocols to harmonize analysis and quality control across multiple sites (see http://enigma.ini.usc.edu/protocols/functional-protocols/).

All denoising steps were performed using the eXtensible Connectivity Pipeline-DCAN (XCP-D) software after resampling the images to standard space [19–21]. Denoising included despiking (AFNI’s 3dDespike), the removal of six motion parameters, their temporal derivatives, and the quadratic expansion of the motion parameters and their temporal derivatives. The six translation and rotation head motion traces were band-stop filtered to remove signals between 12.0 and 20.0 breaths-per-minute using a fourth-order notch filter [22]. Physiological signals, including white matter (WM), cerebrospinal fluid (CSF), and global signal (GS), were also regressed out. A bandpass filter was applied to retain signal fluctuations within the frequency range of 0.01–0.1 Hz, which corresponds to low-frequency resting-state activity. The same filter was applied to the confound regressors.

During quality control, one participant was excluded due to inconsistent acquisition (shorter scan length), five participants were excluded due to poor image processing (brain extraction and/or image registration), and an additional five participants were excluded due to motion artifacts (average framewise displacement > 0.25 mm). Additional 2 participants were excluded due to incomplete demographic information (age, sex or time since injury). The final dataset included 144 adolescents [54.9% female; mean (SD) age = 14.96 (1.85) years, mean (SD) time since injury = 21.94 (8.36) days].

### 2.3 Connectome construction and harmonization

Time series were extracted using the 300-region Seitzman parcellation [23], which includes 239 cortical, 34 subcortical, and 27 cerebellar regions, with subcortical and cerebellar regions functionally assigned to cortical networks using the winner-take-all partitioning method based on resting-state connectivity.

9 ROIs (3%) were excluded due to signal dropout. Average within- and between-network FC (i.e., Pearson correlation between ROI pairs) was calculated for the following resting state networks[24]: Auditory, Cingulo-Opercular, Default Mode, Dorsal Attention, Fronto-Parietal, Salience, Somatomotor (Dorsal and Lateral), Ventral Attention, and Visual (see **Supplemental Table 1** for the final set of included ROIs and corresponding functional brain network). The extracted within- and between-network FC values were harmonized for scanner differences using neuroCombat function v1.0.13 in RStudio [7, 10], with age at injury and sex included as covariates. Two ComBat versions were applied for data harmonization, with and without the EB estimation.

### 2.4 Data analysis

Statistical analyses were conducted using R studio v4.4.1. To assess the performance of each harmonization approach (i.e., ComBat harmonization with and without empirical Bayes), the effect of site was evaluated through separate one-way ANOVA models for each within- and between-network connectivity measure. Site effects not reaching the statistical threshold (*p* > 0.05) were interpreted as evidence that site-related variability had been successfully removed. No multiple comparison correction was applied across measures, which provided a more conservative evaluation for the presence of site effects.

The within-scanner consistency was evaluated across connectivity measures before (unharmonized) and after each harmonization approach (before harmonization versus harmonization with EB, and before harmonization versus harmonization without EB) using the intraclass correlation coefficient (ICC). ICC values were interpreted as follows: <0.50 indicating poor consistency, 0.50–0.75 moderate, 0.75–0.90 good, and ≥ 0.90 excellent consistency [25]. Effective harmonization was expected to reduce site-related effects while preserving the within-scanner variability observed prior to harmonization [14].

To evaluate whether ComBat harmonization preserves biological variability associated with age, Pearson correlation was used to assess the relationship between age and the harmonized functional connectivity values before and after each harmonization approach.

Principal component analysis (PCA) was performed using the prcomp function in R, which computes the singular value decomposition of the centered and scaled data matrix to extract orthogonal components representing uncorrelated sources of variance. The first two principal components (PC1 and PC2), accounting for the largest proportion of variance within each harmonization approach, were retained for further analysis. Consistent with the ANOVA procedures described above, these components were subsequently tested to evaluate potential residual site effects before and after the harmonization procedure.

## 3 Results

### 3.1 Presence of site effects

**Figure 1.**
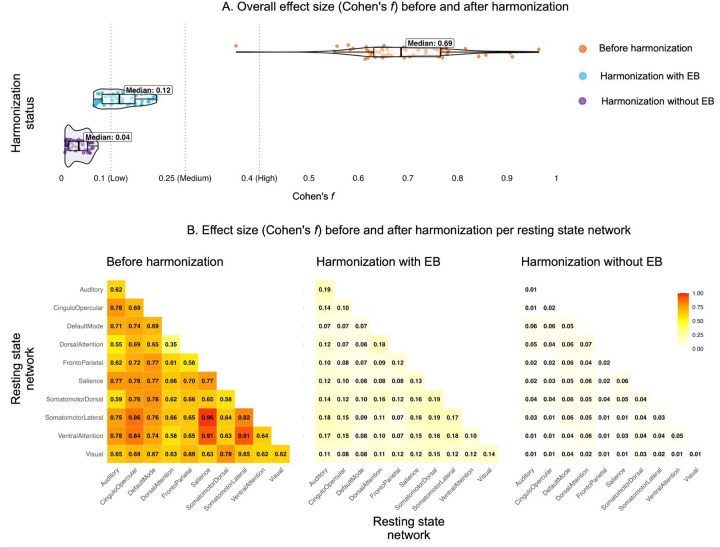
Presence of site effects before and after harmonization (Cohen’s *f* values).

All within- and between-network connectivity measures demonstrated significant site effects before harmonization (Fig. 1 and **Supplementary Table 2**). Effect sizes ranged from moderate to large, with connectivity between the salience and the somatomotor lateral network showing the highest effect (*F*(8, 135) = 15.67, *p* < 0.001, Cohen’s *f* = 0.964), and within the dorsal attention network the lowest [*F* (8, 135) = 2.10, *p* < 0.05, Cohen’s *f =* 0.353]. Following ComBat harmonization, no site effects remained significant, and effect sizes were low for both harmonization approaches, although harmonization with EB demonstrated larger effect sizes [mean Cohen’s *f* (SD) = 0.12 (0.04)] compared to harmonization without EB [mean Cohen’s *f* (SD) = 0.03 (0.02)].

### 3.2 Within-site consistency

**Figure 2.**
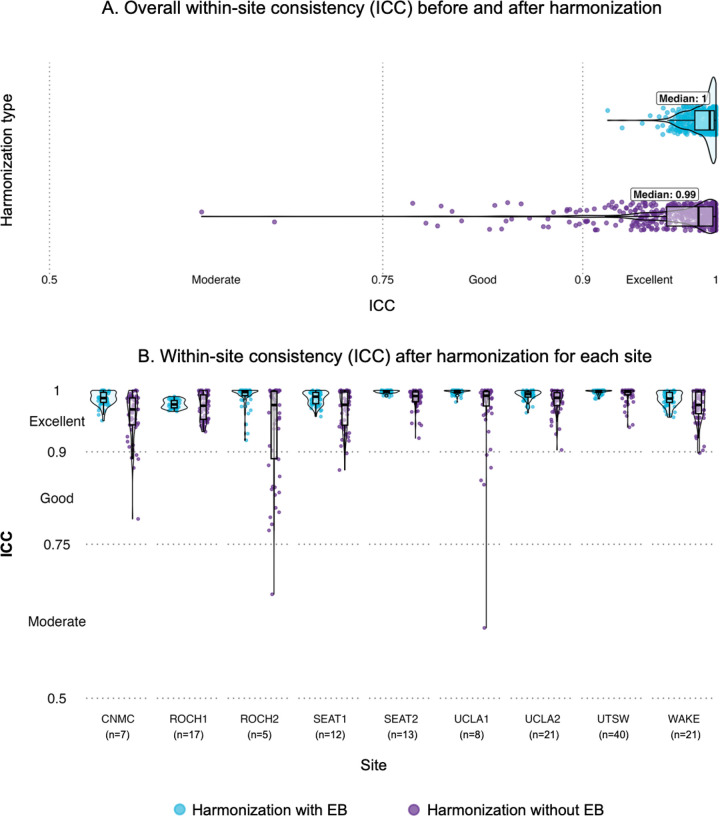
**(A)** Within-site consistency (ICC) between functional connectivity measures before and after harmonization. Higher values mean that within-site variability is preserved following harmonization; **(B)** Within-site consistency across functional connectivity measures by site.

Harmonization with EB demonstrated excellent within-site consistency across all resting state networks and sites [mean (SD) = 0.99 (0.01)]. Moderate to excellent consistency was observed following harmonization without EB (Fig. 2; **Supplementary Fig. 1**), ranging from 0.61 to 1 [mean (SD) = 0.97 (0.04)].

### 3.3 Correlation between functional connectivity and age

**Figure 3.**
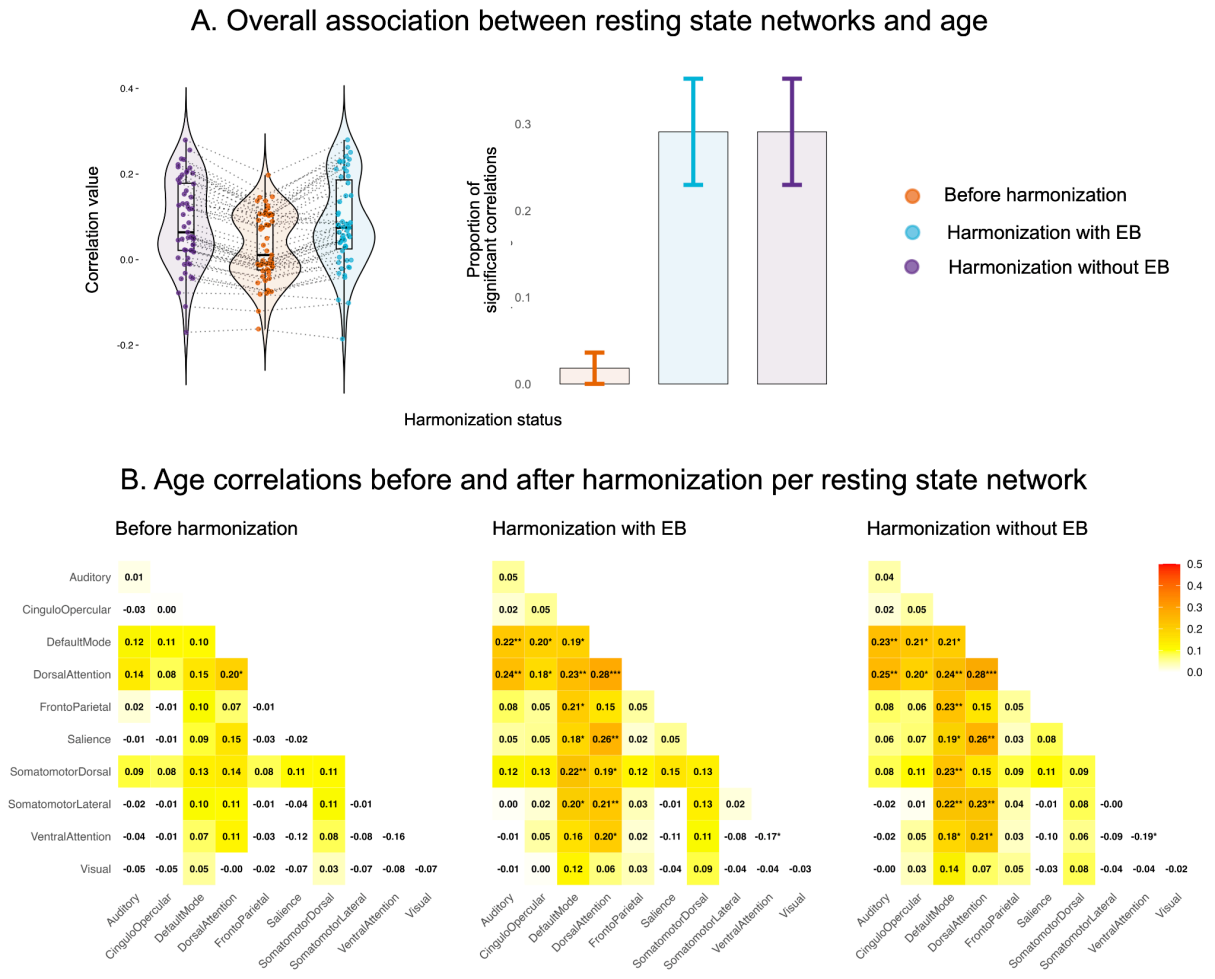
Association between resting state networks and age.

Age-correlation improved following harmonization with and without EB compared to the data before harmonization (Fig. 3, A). The proportion of resting state networks significantly correlated with age increased from 0.02 before harmonization, to 0.29 after either of the harmonization approaches, with patterns of increased correlations present especially among the within and between-network connectivity involving default mode and dorsal attention networks (Fig. 3, B).

### 3.4 Presence of site effects in the principal components

**Figure 4.**
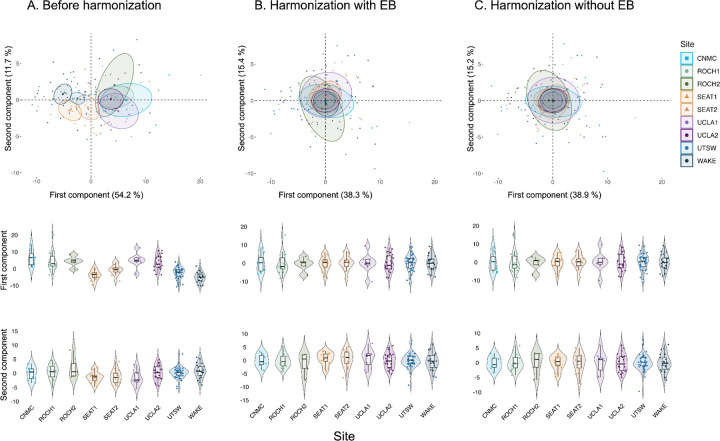
Distribution of the first two principal components before and after harmonization.

The first principal component identified before harmonization explained 54.2% of the variance and showed significant site effects (*F*(8, 135) = 17.70, *p* < 0.001, Cohen’s *f* = 1.02). The second component explained 11.7% of the variance and showed no significant site effects (*F*(8, 135) = 2.00, *p* > 0.05, Cohen’s *f* = 0.34). Following harmonization with EB, no site effects were present in the first (*F*(8, 135) = 0.02, *p* > 0.05, Cohen’s *f* = 0.03) or the second principal component (*F*(8, 135) = 0.33, *p* > 0.05, Cohen’s *f* = 0.14). Site effects were also below significance level for the first (*F*(8, 135) = 0.02, *p* > 0.05, Cohen’s *f* = 0.04) and second principal components (*F*(8, 135) = 0.01, *p* > 0.05, Cohen’s *f* = 0.02) following harmonization without EB. Both harmonization approaches explained less variability based on the first component (38.3% after harmonization with EB and 38.9% after harmonization without EB), but more variability based on the second principal component (15.4.3% after harmonization with EB and 14.2% after harmonization without EB) compared to the data before harmonization (Fig. 4).

## 4 Discussion

The increasing availability of large-scale fMRI datasets in pediatric concussion offers unprecedented opportunities to examine brain function during critical developmental periods. In this multi-site study of concussed adolescents, we investigated the impact of using Empirical Bayes (EB) estimation within the ComBat harmonization framework on within and between-network resting-state functional connectivity measures. To our knowledge, this is the first study to apply ComBat harmonization specifically to within and between-network functional connectivity measures, and to assess the effect of EB estimation for neuroimaging data.

ComBat harmonization was effective in reducing site effects regardless of whether EB estimation was used. This is consistent with past work showing that ComBat provides efficient means of data harmonization for neuroimaging features [7, 9, 13, 14, 26, 27]. However, subtle differences were observed depending on the version of ComBat applied. When using EB estimation, the effect size of the residual site variability was slightly elevated compared to ComBat without EB, although the persistence of site effects did not reach statistical significance following either harmonization approach. Possibly, EB option’s attempt to balance the variability across features with variability across sites retains subtle residual site-related variability. However, since the residual variability associated with site did not reach statistical significance, it is unlikely that the use of EB estimation could meaningfully bias group-level statistical analyses when pooling information across features.

Data harmonization approaches can encounter validation challenges. In the absence of an established ground truth for functional connectivity, we evaluated the extent to which harmonization preserved original data variability by looking at whether site-specific variability is maintained following harmonization. The current results are consistent with prior work examining how ComBat harmonization alters within-site variability in structural connectivity [14], showing moderate-to-excellent ICC when harmonizing graph theory derived from structural imaging without EB. In the current study, within-site consistency was consistently excellent when harmonizing with EB, indicative of better preservation of the original variability in functional brain networks even across sites with smaller sample sizes (n ≤ 12). This is relevant in developmental cohorts and individuals with TBI where interindividual variability can be pronounced, and suggests that EB estimation may be more appropriate when using within- and between-network connectivity measures. It has been indicated that EB estimation would be suitable when the number of features is high relative to the sample size [9]. However, our results suggest that EB estimation can successfully remove site effects, while also maintaining the original variability to a greater extent than harmonization without EB. This effect was prominent across sites with low sample size (see Fig. 2, B **and Supplementary Fig. 2**), enforcing the idea that EB estimation can obtain better estimates for small sample sizes [7, 8]. Averages of functional connectivity measures across brain networks tend to distribute similarly and may show relatively higher correlation compared to other features derived from structural imaging, thus EB harmonization might still be appropriate when the number of features is relatively smaller. Future studies should address how the similarity between features can affect the efficiency of harmonizing with and without EB estimation for different neuroimaging measures and modalities, and evaluate the persistence of site effects versus within-site consistency in the context of similarity across features.

An overall improvement in the correlation between functional brain networks and age was present in the harmonized networks regardless of whether EB estimation was used. This finding is consistent with prior studies using ComBat harmonization, which have similarly reported enhanced age correlations after adjusting for site-related variability [9, 27]. Such increases are typically interpreted as reflecting improved biological signal fidelity in the harmonized data, whereby true neural variability is more accurately preserved as site-related effects are reduced. Notably, we found that age-related correlations were predominantly strengthened across connections within and between the default mode network and the dorsal attention network. This aligns with developmental neuroimaging research demonstrating that functional connectivity within and between these networks systematically changes with age across childhood and adolescence [28, 29], indicative of brain maturation that correspond to cognitive development and age-related shifts in attention and self-referential processing [30, 31]. Our results thus reinforce the notion that harmonization enhances the ability to detect meaningful age-related neurodevelopmental trajectories by reducing confounding site effects and preserving biologically relevant connectivity patterns.

The first principal component derived based on within- and between-network connectivity measures demonstrated site effects to a higher extent than the second component (Fig. 4), suggesting that site effects constitute a primary source of variability in multisite functional connectivity data. This may obscure meaningful biological variability for example by reducing age-related associations across sites. Site effects were absent from the principal components when PCA was applied on harmonized features following either harmonization approach, consistent with a previous study showing reduced site-dependent clustering following ComBat harmonization in structural MRI [13]. The presence of site effects in the first principal component before harmonization raises the possibility that sources of multisite variability could be present in the (multivariate) relationships across features. The EB estimation should be beneficial for the removal of site effects within principal components by pulling information across features. However, harmonization with EB estimation did not provide a clear advantage in controlling site-related artifacts in the principal components (see Fig. 4). This evaluation may be particularly relevant for studies addressing research questions that involve multivariate group-analysis, where the relative weighting of different features can be important when interpreting the results. Future studies might address whether the use of EB estimation provides advantages regarding how the relative weighting of different features is preserved. In addition, the proportion of explained variance decreased for the first component, but increased for the second component following either ComBat harmonization with or without EB. This potentially reflects more subtle biological variability that was previously overshadowed by batch effects and might have impacted the second component even though the first component already contained most of the variance associated with site effects. It also suggests that applying ComBat harmonization before, rather than after PCA can provide more meaningful components.

Several limitations should be considered. Although ComBat harmonization effectively reduced site effects in the current sample, the generalizability of these results to other cohorts, scanner models, or acquisition protocols remains to be confirmed. We focused on within- and between-network connectivity measures, however, other functional connectivity estimates commonly used in TBI research should be evaluated in future studies. In addition, average within- and between-network connectivity features can be correlated, and features derived using other imaging modalities may show different correlation structure and may distribute differently post-harmonization depending on the use of EB estimation. Future studies using other rs-fMRI features or different imaging modalities should consider undergoing independent analyses to confirm the generalizability of the current findings. The efficiency of ComBat harmonization and the impact of EB estimation in datasets spanning broader age ranges, injury severities, and post-injury time points should be considered.

Our results suggest a modest trade-off between fully removing site effects and preserving within-site, biologically meaningful variability. Both harmonization approaches provided effective means for removing site effects, with each offering distinct advantages. Standard ComBat with EB estimation better preserved original variability within sites, while ComBat without EB provided slightly stronger harmonization by more aggressively removing site effects, but at the cost of reducing within-site consistency. Future work should continue to explore strategies that balance these goals, particularly in large-scale pediatric neuroimaging studies where developmental variability is a key concern.

## Supplementary Material

This is a list of supplementary files associated with this preprint. Click to download.

• SupplementaryMaterial.docx

## Figures and Tables

**Table 1:** Descriptive statistics across sites.

Site	n*** (%)	Sex [*n* (% female)]	Age [Mean (SD)]	DSI** [Mean (SD)]
CNMC	7 (4.9%)	6 (85.7%)	15.26 (2.15)	22.57 (8.92)
ROCH1	17 (11.8%)	7 (41.2%)	14.37 (2.15)	24.24 (9.07)
ROCH2	5 (3.5%)	4 (80%)	13.14 (2.03)	26 (8.12)
SEAT1	12 (8.3%)	8 (66.7%)	14.64 (1.83)	22.25 (7.29)
SEAT2	13 (9%)	10 (76.9%)	15.53 (1.96)	18.92 (9.6)
UCLA1	8 (5.6%)	3 (37.5%)	15.39 (1.66)	29.25 (5.34)
UCLA2	21 (14.6%)	10 (47.6%)	15.1 (1.71)	25.48 (8.27)
UTSW	40 (27.8%)	19 (47.5%)	14.98 (1.65)	17.88 (6.46)
WAKE	21 (14.6%)	12 (57.1%)	15.24 (1.93)	22 (8.38)

The final dataset included 144 adolescents [54.9% female; mean (SD) age = 14.96 (1.85) years, mean (SD) time since injury = 21.94 (8.36) days]. DSI - days since injury. Kruskal-Wallis tests were used for continuous variables, and Chi-square tests were used for categorical variables.

* p< 0.05

** p< 0.01,

*** p < 0.001

## Data Availability

Data analysis code used in this study is available is the following link: https://anonymous.4open.science/r/C4K_combat_analysis-3D38. Due to data-sharing restrictions, raw data are not publicly available and can be accessed only through the Federal Interagency Traumatic Brain Injury Research (FITBIR) Informatics System (https://fitbir.nih.gov). Qualified researchers may request access to the raw data via FITBIR, subject to appropriate data use agreements and approvals.
